# Identification of ejaculated proteins in the house mouse (*Mus domesticus*) via isotopic labeling

**DOI:** 10.1186/1471-2164-12-306

**Published:** 2011-06-10

**Authors:** Matthew D Dean, Geoffrey D Findlay, Michael R Hoopmann, Christine C Wu, Michael J MacCoss, Willie J Swanson, Michael W Nachman

**Affiliations:** 1 Molecular and Computational Biology, University of Southern California, 1050 Childs Way, Los Angeles, CA, USA; 2 Ecology and Evolutionary Biology, University of Arizona, Tucson, AZ, USA; 3 Department of Genome Sciences, University of Washington, Seattle, WA, USA; 4 Department of Cell Biology, University of Pittsburgh, Pittsburgh, PA, USA

**Keywords:** seminal fluid, ejaculate, evolution

## Abstract

**Background:**

Seminal fluid plays an important role in successful fertilization, but knowledge of the full suite of proteins transferred from males to females during copulation is incomplete. The list of ejaculated proteins remains particularly scant in one of the best-studied mammalian systems, the house mouse (*Mus domesticus*), where artificial ejaculation techniques have proven inadequate. Here we investigate an alternative method for identifying ejaculated proteins, by isotopically labeling females with ^15^N and then mating them to unlabeled, vasectomized males. Proteins were then isolated from mated females and identified using mass spectrometry. In addition to gaining insights into possible functions and fates of ejaculated proteins, our study serves as proof of concept that isotopic labeling is a powerful means to study reproductive proteins.

**Results:**

We identified 69 male-derived proteins from the female reproductive tract following copulation. More than a third of all spectra detected mapped to just seven genes known to be structurally important in the formation of the copulatory plug, a hard coagulum that forms shortly after mating. Seminal fluid is significantly enriched for proteins that function in protection from oxidative stress and endopeptidase inhibition. Females, on the other hand, produce endopeptidases in response to mating. The 69 ejaculated proteins evolve significantly more rapidly than other proteins that we previously identified directly from dissection of the male reproductive tract.

**Conclusion:**

Our study attempts to comprehensively identify the proteins transferred from males to females during mating, expanding the application of isotopic labeling to mammalian reproductive genomics. This technique opens the way to the targeted monitoring of the fate of ejaculated proteins as they incubate in the female reproductive tract.

## Background

Successful fertilization occurs through complex interactions among a diversity of proteins that mediate the final fusion of male and female pronuclei. In internally fertilizing species, sperm are accompanied by a non-sperm component of seminal fluid that functions in a variety of contexts. In mammals, this seminal fluid derives from several compartments of the male reproductive tract, the experimental removal of which leads to reductions in fertility success [1,2], smaller litter sizes [3] and delays in oocyte penetration and embryonic development [4-6]. Seminal fluid also influences sperm motility and physiological status [7-11], suppresses the female immune system [12-14], protects sperm from neutrophil attack in the female reproductive tract [15,16], prepares the uterus for implantation [17], and alters female mating behavior [18,19]. In insects, seminal fluid induces egg laying and proper sperm storage [20-24] and mediates sperm competition outcomes [25-30].

Some properties of ejaculated proteins suggest they may be a source of sexual conflict. In many animal species, including worms [31,32], insects [33], reptiles [34-36], and mammals [37-40], ejaculated proteins coagulate to form a copulatory plug (also referred to as a mating plug or vaginal plug). By blocking access to the uterus and oviducts, the plug is thought to be an adaptation by which males inhibit the passage of sperm from competitor males, thus protecting their reproductive investment. This hypothesis predicts that the copulatory plug is on average deleterious to females because it inhibits future mate choice. In mice, the copulatory plug is probably effective at inhibiting sperm from other males, because it remains intact for approximately 24 hours, females are truly fertile for about 4-12 hours during the estrus cycle, and sperm are not stored across estrus cycles [40]. Nevertheless, multiple paternity is still common [41,42]. Species which do not form copulatory plugs usually show alternative means of mate-guarding, or have mating ecologies that tend towards monogamy where mate-guarding would be unnecessary [38,40]. However, some apparently monogamous species of rodents like *Peromyscus polionotus*, in which sexual conflict is expected to be less severe, also form a copulatory plug [43].

Additional hypotheses for the function of the copulatory plug include male-female signaling necessary for proper implantation of embryos. For example, copulatory stimulation is necessary to prime the female uterus for implantation [44,45], and the plug may function in this context. The hypothesis that the plug prevents leakage of semen is inconsistent with experiments showing that removal of the plug does not inhibit fertilization, pregnancy, or parturition [46,47]. Similarly, the hypothesis that the plug acts as a reservoir regulating the release of sperm [48] is inconsistent with plug transfer experiments in guinea pigs [47].

A better understanding of the functions of seminal fluid requires a fuller picture of the proteins that are transferred from males to females in the ejaculate. Using house mice (*Mus domesticus*) as a model system, we mated vasectomized males to females that had been metabolically labeled with a heavy isotope of nitrogen, ^15^N. We then used mass spectrometry to identify unlabeled, ejaculated proteins directly from the female reproductive tract. We identified 69 ejaculated proteins from female reproductive tracts 6-14 hours post-coitus. Using current functional annotations, we showed that seminal fluid was significantly enriched for genes that participate in two main processes: protection from oxidative stress and endopeptidase inhibition. We also found that more than a third of all identified spectra mapped to just seven proteins known to form the copulatory plug, suggesting a large portion of the ejaculate is dedicated to the formation of this structure. By comparing mated to unmated females, we found that females produced endopeptidases in response to mating. Interestingly, the 69 ejaculated proteins were a non-random subset of the ~500 proteins that we previously identified directly from dissected regions of the male reproductive tract [49]. The ejaculated proteins we detected here evolved significantly more rapidly than the other male reproductive proteins. These patterns are consistent with the hypothesis that sexual selection has driven the evolutionary dynamics of ejaculated proteins. Future testing of this hypothesis is made possible by the techniques implemented here.

## Methods

### Mice used

Breeding and genotypes followed Dean et al. [49]. We generated F1 progeny from crosses between two different wild-derived inbred strains of *Mus domesticus *(female LEWES/EiJ x male WSB/Eij). F1 mice were then mated with each other to identify proteins transferred during mating. F1 mice were used rather than fully inbred strains to avoid the deleterious effects of inbreeding. We paired parental female LEWES/EiJ mice with male WSB/EiJ mice for one week, then separated them so the dam gave birth in isolation. At 21 days postpartum, F1 males were weaned individually, and F1 females were weaned in groups. Males were weaned individually because grouped males have comparatively reduced fertility [50], probably due to suppression by dominant males. F1 females labeled with ^15^N (see below) were then mated to unlabeled, vasectomized F1 males. All husbandry and experimental manipulations were approved by the University of Arizona Institutional Animal Care and Use Committee.

We measured the size of copulatory plugs in an additional set of mice derived from wild parents trapped more than 100m apart around Tucson, AZ, USA and then crossed in the laboratory. Wild derived F1 males were then mated to a common female genotype (F1 of female LEWES/EiJ x male WSB/Eij crosses). In total, copulatory plugs were measured from 149 crosses from 47 different F1 males, derived from 9 wild caught sires and 15 wild caught dams.

### Isotopic labeling of females

Artificial ejaculation techniques such as electroejaculation produce abnormal and inconsistent ejaculates in mice [51,52], so we instead employed isotopic labeling to differentiate male- and female-derived proteins [53]. ^15^N-enriched diets were prepared by combining ^15^N-labeled *Spirulina platensis *(>99 atom percent excess, Spectra Gases Inc., now part of Cambridge Isotope Laboratories, Inc., Andover, MA) with protein-free rodent diet (TD 93328, Harlan, Indianapolis, IN) in a 1:2 (mass:mass) ratio as previously described [54,55]. The two food types were ground into a homogenous powder with a mortar and pestle and worked into a dough by slowly adding water (roughly 5-6 ml water/30 grams powder mixture). The dough was formed into 1.5 cm^3 ^pellets and placed in a food dehydrator set at 54°C until completely dry.

Three-week-old females were weaned from their mothers and immediately given ^15^N-enriched diet. In contrast, all males used in this experiment were fed regular diet. Female proteins will have a shifted mass as a result of incorporation of ^15^N. To gauge the effectiveness of our labeling strategy, we analyzed two non-reproductive tissues from a mated female: the liver, an organ with a relatively high rate of protein turnover, and the brain, which has a low rate of protein turnover. Under unlabeled search conditions, we identified five proteins from the liver and 103 proteins from the brain. These data confirmed that 15N labeling more effectively inhibited identification of female-derived proteins in tissues with faster protein turnover. As discussed below (*Analyzing an unmated female*), the low number of unlabeled proteins identified from the unmated female reproductive tract appears more similar to the high turnover liver tissue, suggesting that our labeling strategy was effective in masking female-derived proteins to enable detection of ejaculated proteins.

### Vasectomization of males

Males approximately eight weeks of age were anesthetized with 2.5% avertin, then vasectomized using standard techniques [56]. We used vasectomized males because we were interested in the seminal fluid proteins and wanted to exclude the sperm proteome, which is complex [57-60]. Males of this genotype are sexually mature by eight weeks of age [61]. Cuts were closed using surgical clips and males were checked several times a day to monitor recovery. One week after vasectomy, clips were removed. One week following clip removal, males were mated to tester females that had been induced to ovulate using standard techniques [56,62]. These test matings confirmed libido and the absence of sperm in dissected female reproductive tracts. Males were mated to tester females in consecutive weeks; vasectomized males were mated to at least three tester females prior to mating with labeled females. In total, two vasectomized males were analyzed in the present study.

### Mating and collection of samples

After three to four weeks of feeding on ^15^N chow, labeled females were induced to ovulate using standard techniques [56,62]. Immediately following administration of the hormone hCG, labeled females were paired with vasectomized males. Between 12 and 20 hours after initial pairing (likely to be 6-14 hours after mating), females were sacrificed and reproductive tracts were removed. Internal fluids were stripped from both uteri and immediately frozen at -80°C, as were the copulatory plug, the remaining reproductive tract, the brain, and the liver. As a control, we collected a reproductive tract, brain, and liver from a labeled female that was exposed to a male but had not mated. In total, proteins from two mated females and one unmated female were analyzed with mass spectrometry.

### Protein preparation and mass spectrometry

As a result of labeling, female-derived proteins were expected to have upward-shifted masses, making it possible to distinguish male- and female-derived proteins sampled from mated female reproductive tracts. Samples were generally prepared and analyzed by mass spectrometry as previously described [49,53] with some modifications. Tissue samples (dissected female reproductive tracts, liver, brain) were homogenized in 50 mM ammonium bicarbonate. The homogenate was centrifuged at 20,800 *g *for 5 min, and the soluble fraction was retained. Soluble proteins were quantified with a BCA assay (Thermo) and then mixed with PPS detergent (Protein Discoveries) to a final concentration of 0.1% PPS. Proteins were denatured, reduced and alkylated as described previously [63] and then digested with trypsin. PPS was hydrolyzed by the addition of HCl to a final concentration of 200 mM. Copulatory plugs were processed by placing slices of plug in 50 mM ammonium bicarbonate with 0.1% PPS and then sonicating 10 times with a probe sonicator, alternating 45 seconds of sonication with 45 seconds of ice incubation. Plug samples were then boiled for 2 min and homogenized with a pestle homogenizer. A few seconds of microcentrifugation removed remaining large pieces of solid plug, and the remaining, cloudy supernatant was then reduced, alkylated and trypsin digested as above.

Tryptic peptides of all samples were separated using 75-μm internal diameter fused silica HPLC columns packed with 35 cm of Jupiter C12 (4 μm, 90 Å; Phenomonex) reversed phase material. These columns were placed on-line with a LTQ-FT Ultra mass spectrometer (Thermo), and peptides were eluted over a 3-hour gradient. For each sample analyzed, we ran 5-7 technical replicates, each loading ~5 μg protein onto the column. Except as described below ("*Accurate mass-directed tandem mass spectrometry*"), mass spectra were obtained using data-dependent acquisition. We focused on four biological samples - two different copulatory plugs and two different uterine fluid samples isolated from two different matings - for analyses of reproductive proteins (Additional File 1).

In making protein identifications from the collected MS data, we purposely set our identification criteria to have a high false negative and low false positive rate to lend confidence to protein identifications. MS2 files from each experiment were searched against two databases using the SEQUEST algorithm [64]: one database contained all proteins from the NCBI build 37 mouse genome, while the other contained randomly shuffled protein sequences representing decoy proteins. Results from these searches were analyzed with the PERCOLATOR program [65,66] to improve discrimination between correct and incorrect peptide-spectrum matches and to set a per-spectrum false discovery rate (FDR) of 0.01. However, previous research has shown that with a per-spectrum FDR of 1%, the peptide and the protein-level FDR can be much higher [8-11%, depending on the search algorithm used, 67]. Most of these false positive protein identifications were presumably those proteins identified with a single peptide. Thus, to consider a protein identified in this study, we required it to have been matched by at least two peptides, at least one of which was a unique match to a single region in the genome.

### Normalized Spectral Abundance Factor (NSAF)

It is difficult to relate spectral counts to protein abundance because not all peptides within proteins are equally identifiable [68]. The acquisition of tandem mass spectrometry data is a semi-random process and is highly dependent on the presence of co-eluting molecular species. Signal suppression during electrospray ionization can potentially alter the mass spectrometry signal response within complex mixtures. Longer proteins may be more detectable simply because they are more likely to contain tryptic and ionizable peptides. Post-translational modifications such as glycosylation may further hinder identification of unmodified proteins.

Nevertheless, more abundant proteins should have a greater number of spectra mapping to their sequence compared to low abundance proteins [69,70]. As a rough proxy of relative protein abundance, we calculated the normalized spectral abundance factor (NSAF) [69,70], with some slight modifications. Here, we calculated a single experiment-wide NSAF for each gene by summing all spectral counts across the four main biological samples (two copulatory plugs, two uterine fluid samples), dividing this sum by the protein length, then dividing by the sum of this value across all genes. NSAF therefore ranges from 0 to 1 for each protein (actual observed range = 10^-5 ^to 0.21, median = 0.002) and sums to 1 across all 69 identified proteins. Relatively high NSAF may indicate higher abundance in the sample, though the caveats discussed above suggest cautious interpretation.

for genes that encoded multiple alternative transcripts, we divided by the median transcript length; our results did not change if instead we divided by the shortest, the longest, or a randomly chosen transcript length. Our results also did not change if we calculated NSAF separately for each of the four biological samples; we present the experiment-wide NSAF for simplicity. For spectra that mapped to more than one region of the genome, we divided the number of spectra by the number of regions it mapped to, adding the result to each gene's spectral count. However, as described above, a gene was only considered present if at least two different peptides mapped to it, at least one of which was a unique hit to that gene product.

For comparison, we re-analyzed the proteins identified from dissected regions of the male reproductive tract [49]. We calculated NSAF as described above, summing spectral count across the six distinct regions of the male reproductive tract sampled.

### Evaluating Detection Sensitivity

Three targeted searches provided support that we identified most detectable ejaculated proteins. These three methods of evaluating detection sensitivity suggested that additional technical and/or biological replicates would not have yielded a substantially larger list of ejaculated proteins under the experimental conditions employed here.

#### Isolating insoluble proteins

In an attempt to detect male-derived proteins that could be bound to the female epithelium, we ran five technical replicates on the insoluble fraction of one of the mated female's reproductive tract. We isolated insoluble proteins by resuspending the pellet from centrifugation in 0.5% PPS and then sonicating twice with a probe sonicator.

#### Depletion of highly abundant proteins

In an attempt to unmask less abundant proteins, we re-analyzed one of the copulatory plug samples and one of the uterine fluid samples after depleting each of them of highly abundant immunoglobulin- and albumin-like proteins. We used the ProteoPrep ImmunoAffinity Albumin and IgG Depletion Kit (Sigma) to reduce levels of albumin and IgG proteins.

#### Accurate mass-directed tandem mass spectrometry

We also used an analytical method to direct the mass spectrometer to specifically fragment male-derived peptides that had not been previously sampled in a prior technical replicate [71]. We re-analyzed one of the plug samples and one of the uterine fluid samples, first running one technical replicate using data-dependent acquisition. We then used the HARDKLÖR algorithm [71,72] to identify peaks from MS1 signals that were predicted to come from a peptide with a natural abundance isotope distribution (i.e., an unlabeled male peptide). We constructed a list of these peptides' *m*/*z *(+/- 10 ppm) and elution times (± 1.5 min) off of the HPLC column and used this list to direct the mass spectrometer's peptide sampling for two subsequent technical replicates. If no peptides on the list were detected at a given elution time, the instrument used standard data-dependent acquisition to sample peptides from that MS1 scan. Finally, we compared the number of proteins and peptides identified from three technical replicates that used this method to the number identified by three standard, data-dependent technical replicates performed on the same samples.

### Testing for functional overrepresentation

We took two approaches to identify important functions in ejaculated proteins. First, we tested for statistical enrichment of genes with particular Gene Ontology functional annotations [73], using ONTOLOGIZER version 2.0 [74], with the "Term-for-Term" calculation method and Bonferroni-corrected P < 0.05. Among the 69 ejaculated proteins, 68 could be linked to Gene Ontology data. Second, we qualitatively examined genes to look for commonality of function among proteins with high NSAF.

### Analyzing female-derived proteins

#### Analyzing an unmated female

As a negative control, we attempted to identify unlabeled proteins from a female that had undergone ^15^N labeling for three weeks and was paired with a male for approximately 20 hours, but where copulation did not take place, as confirmed by the absence of a copulatory plug. In theory, we should not identify unlabeled proteins unless i) certain proteins fail to incorporate ^15^N, for example proteins with low rates of turnover, or ii) the male mounted and transferred some proteins without true ejaculation. We identified two large hemoglobin families, an actin family, and *SVS4 *when searching mass spectra from this virgin female's reproductive tract under the assumption of naturally occurring isotope distributions. The hemoglobin and actin families could plausibly be explained by their apparently high abundance - by chance we may have sampled a few relatively unlabeled peptides. Identification of *SVS4*, from five spectra derived from two uniquely mapping peptides, was surprising because this is a quintessential seminal vesicle secretion that is derived from the male reproductive tract. It is possible that mounting without ejaculation occurred and some male proteins were transferred at a low level. Notably, unlabeled SVS4 was identified with roughly two orders of magnitude more spectra from mated females, suggesting the SVS4 identified in the virgin female was an anomaly and that this is truly a male-transferred protein.

#### Labeled protein searches

Although this experiment was specifically designed to identify ejaculated proteins, we also identified female-derived proteins that could be induced from mating. We performed SEQUEST searches in which we adjusted the search parameters to find proteins that were labeled with 95% ^15^N incorporation. Specifically, we altered the SEQUEST search parameters such that the expected molecular mass of each amino acid was increased by (0.95 Daltons) x (the number of nitrogen atoms in the amino acid), which corresponds to an expected 95% labeling. We analyzed the two copulatory plug samples in this manner. Because the SEQUEST algorithm allows some deviation between the theoretical mass of a peptide and the mass observed by the mass spectrometer, assuming an additional mass of 0.95 Daltons/nitrogen atom would not necessarily preclude identification of labeled proteins with similar levels of ^15^N incorporation (e.g., 92% labeled peptides may still be identified).

### Estimating evolutionary rate and adaptive evolution

We analyzed pairwise *d*_N_/*d*_S _estimates of all genes in the genome that have one-to-one orthologs between mouse and rat, taken from Dean et al. [49]. Briefly, all orthology assignments and sequences were downloaded from Ensembl version 48, NCBI mouse build 37 (http://www.ensembl.org). Protein sequences were aligned using CLUSTALW version 1.83 [75], associated with their coding DNA sequences using REVTRANS version 1.5 [76], and *d*_N_/*d*_S _estimated using the method of Goldman and Yang [77] as implemented in PAML version 3.15 [78]. We removed any genes with fewer than 100 aligned codons, an estimated *d*_N_>1, or an estimated *d*_S_≥0.381 as quality control measures [details in 49]. We analyzed the full genome in this manner.

Tests for recurrent positive selection were also taken from Dean et al. [49], who analyzed evolutionary rates across five species with the phylogeny of ((mouse, rat), human, (dog, cow)). Briefly, a gene was considered to have experienced a history of recurrent adaptive evolution if five criteria were met: 1) the data fit the M8 model significantly better than M7 at P < 0.01 [79], 2) the data fit the M8 model significantly better than M8a at P < 0.01 [80], 3) the additional class of *d*_N_/*d*_S _estimated by M8 was greater than 1.1, 4) at least 1% of the codons belonged to this additional class of *d*_N_/*d*_S_, and 5) Fixed Effect Likelihood (FEL) analyses [81] revealed significant evidence of positive selection in at least one codon [dN/dS > 1.1 at P < 0.10, the p-value recommended by 82]. As a quality control measure, we excluded any genes whose pairwise *d*_S _exceeded twice the genome median across any of the pairwise combinations of species [details in 49]. We analyzed the full genome in this manner.

## Results

### Identification of ejaculated proteins from the female reproductive tract

We directly identified ejaculated proteins from four biological samples: the two copulatory plugs and two samples of the uterine fluids, from two different male-female matings. The costs associated with isotopic labeling inhibited additional sampling. We considered a gene to be positively identified if at least two different peptides mapped to it, at least one of which mapped uniquely to a single location in the genome. With these criteria, we identified 69 genes total (Additional File 1) from 27,565 spectra representing 827 different peptides, 795 of which mapped to a single location in the genome. Each gene was identified with a median of 80 spectra, seven different peptides (a median six of which mapped uniquely to that gene), at a median coverage of 21.4% of the protein. The median number of spectra per gene is ~ four times lower than the mean number of spectra per gene ( = 399 spectra), indicating that a relatively few genes were identified with a high number of spectra. Genome duplications and high relatedness among certain gene families prevented some gene identifications because associated peptides did not map to a single genomic location. These ambiguous gene identifications are not considered further here but are presented in Additional File 2.

### Evaluating detection sensitivity suggests most detectable proteins were identified

**Figure 1:**
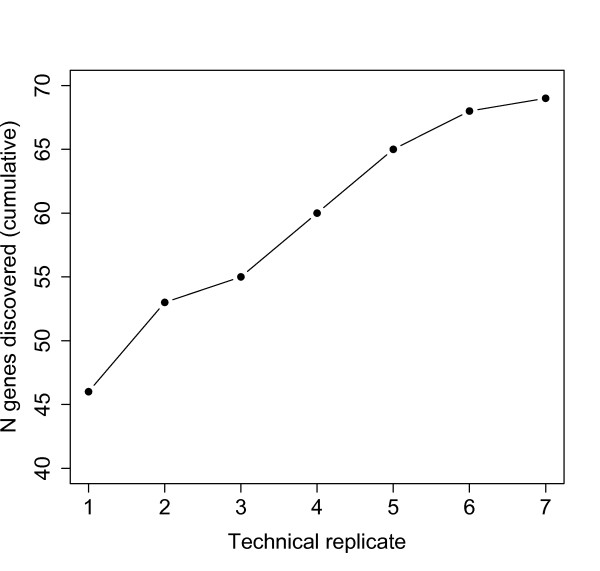
**A cumulative distribution showing new genes discovered across technical replicates**. The sixth and seventh technical replicate added a combined total of four new genes (out of 69 total), suggesting we have approached an asymptote of new gene discovery.

**Figure 2:**
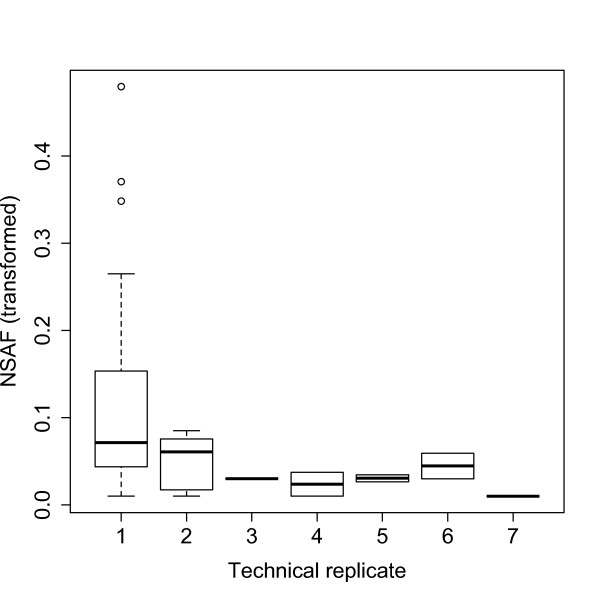
**Genes discovered in the first replicate had higher NSAF (arcsin square root transformed) than genes discovered in later replicates**. This pattern was seen in all four biological samples; we present one of the copulatory plug samples here. This result suggests that we have identified all reasonably abundant (and detectable) proteins under the experimental conditions employed.

Technical replication verified that most detectable proteins were identified under our experimental conditions. The two uterine samples were each run through five technical replicates, and the two plugs were each run through seven technical replicates. Only four additional proteins were identified in the sixth and seventh plug replicates combined (Figure 1). Furthermore, proteins identified for the first time in later technical replicates showed lower median NSAF (Figure 2), suggesting most proteins that were reasonably abundant (and detectable) had been sampled.

Three targeted searches provided additional evidence that we identified most detectable ejaculated proteins. First, we isolated insoluble proteins from the female reproductive tract. In this insoluble fraction, we identified an additional six proteins that were not identified in any other samples (*POU domain class 4 transcription factor 1, elastin, DEAH box polypeptide 9, AT rich interactive domain 1B, histone cluster 1 H1e*, and *tubulin beta 2c*, identified with 2, 2, 3, 4, 8, and 26 spectra, respectively). Second, we re-analyzed one of the copulatory plug samples and one of the uterine fluid samples after depleting each of them of immunoglobulin- and albumin-like proteins, which were highly represented in early technical replicates. Only four additional proteins were newly detected (*major urinary protein 4, transferrin, aldolase 1 A isoform*, and *cathepsin L*, identified with 2, 2, 3, and 7 spectra, respectively) in depleted samples. Third, we re-ran several experiments after directing the mass spectrometer to only fragment peptides that had previously gone unanalyzed [72]. This directed sampling method had a minimal effect. A median of only 2 additional spectra were detected per gene for the copulatory plug sample, out of a total of 13,299 spectra used to identify 62 genes. For the uterine fluid sample, a median of 7 fewer spectra were detected per gene, out of a total of 9,725 spectra mapping to 50 genes. In sum, our evaluations of detection sensitivity provided support that we have identified the major ejaculated proteins present in the female, at least given the experimental conditions employed here.

### Ejaculated proteins were statistically enriched for genes that protect from oxidative stress and inhibit endopeptidases

Two main branches in the Gene Ontology were significantly overrepresented among the 69 ejaculated proteins compared to the entire genome: *antioxidant activity *and *endopeptidase inhibitor activity*. Both functions were overrepresented among genes identified directly from male reproductive tissues [49]. Both functions were also overrepresented in human ejaculates, revealing commonalities among mammalian ejaculate function [49, their supplementary table 4, 83]. Five ejaculated proteins had *antioxidant activity *(compared to 57 of 14,720 annotated genes across the genome, Bonferroni-corrected P < 0.01). Six ejaculated proteins showed evidence of *endopeptidase inhibitor activity *(vs. 148/14,720 in the genome, Bonferroni-corrected P < 0.02).

### Most spectra map to proteins associated with the copulatory plug

A large proportion of the proteins detected were associated with the copulatory plug. Of the 69 genes identified, 62 were found in the copulatory plug samples. It is thought that the copulatory plug forms via the action of the prostate-derived *transglutaminase 4*, which cross-links proteins of at least six seminal vesicle secretions - *SVS1, SVS2, SVS3a, SVS3b, SVS4*, and *SVS5 *[84-87]. In total, these seven proteins were identified with 10,239 spectra, accounting for 37% of all identifiable spectra generated across the four biological samples (two copulatory plugs, two uterine fluid samples), in spite of the fact that their combined length accounted for only 8% of the combined length of all proteins identified.

To further explore the investment that males make in copulatory plugs, we made 149 crosses from 47 different F1 males derived from wild caught parents. These crosses using wild-caught mice were only used to assess natural variation in the weight of the copulatory plug; all other data in this manuscript were derived from F1 (male WSB/Eij x female LEWES/EiJ) matings as described above. Approximately 12 hours after mating, the copulatory plug weighed a median 31 mg, which represented approximately 0.3% of the body weight of the females from which these plugs were collected. We corrected by female weight as a rough proxy for the size of the vaginal-cervix canal, which may constrain the size of the plug. By comparison, a single testis from the male mice that formed these plugs accounts for a median 0.5% of its body mass, suggesting the plug represents a significant investment for males.

### Female-derived proteins

To demonstrate another potential application of the differential labeling method, we identified ^15^N-labeled (presumably female-derived) proteins by computationally adjusting the SEQUEST search algorithm to assume 95% ^15^N incorporation into peptides. Three additional criteria facilitated identification of female-derived proteins that were indeed produced in response to mating. We required female-derived proteins to i) have a secretion signal at P > 0.90, as predicted by TargetP [88], ii) not be identified from an unmated ^15^N-labeled female reproductive tract, and iii) not be identified as a male-derived seminal fluid gene. Using these criteria, we identified six female-derived proteins produced in response to mating - *lactotransferrin *(54 spectra, 14 peptides), *kallikrein-related peptidase 14 *(14 spectra, 3 peptides), *lipocalin 2 *(32 spectra, 2 peptides), *chloride channel calcium activated 3 *(65 spectra, 15 peptides), *corneodesmosin *(, and *alpha-2-HS-glycoprotein *(6 spectra, 2 peptides). Two of these proteins (*lactotransferrin *and *kallikrein-related peptidase 14*) included domains indicative of endopeptidases [89-91], which are proteins that cleave other proteins.

### The 69 ejaculated proteins identified were a non-random subset of proteins produced in the male reproductive tract

Previously [49], we identified 506 proteins from six distinct regions of the reproductive tract - seminal vesicles, anterior prostate (a.k.a. the coagulating gland), ventral prostate, dorsolateral prostate, bulbourethral diverticulum, and the bulbourethral gland (a.k.a. Cowper's gland) - from the same genotype analyzed here (an F1 male derived from a cross between a male WSB/Eij and a female LEWES/EiJ). We re-analyzed those data with the same criteria presented above, producing a list of 483 total single-region proteins (Additional File 3). We found that 54 genes overlapped between the two studies, while 429 genes that were detected in our previous study of the male reproductive tract were not identified here. For simplicity, we refer to these as the 429 "non-overlapping" proteins. If we required only a single uniquely mapping peptide (rather than requiring at least two peptides mapped, at least one of which was unique), we still only observed 72 of the 483 previously identified proteins.

**Figure 3:**
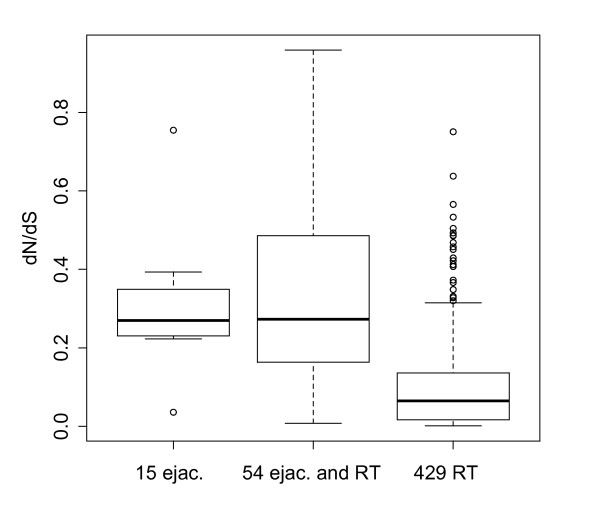
**Genes identified in the present study, including 15 unique to ejaculates (ejac.) and 54 that overlapped with our previous study of the male reproductive tract (RT), evolve significantly more rapidly than the 429 non-overlapping proteins identified in our previous study **[49].

The 54 overlapping genes evolved significantly more rapidly than the 429 non-overlapping genes (Figure 3). Of the 54 overlapping genes, 29 had a one-to-one ortholog in rat and produced estimates of evolutionary rate that satisfied various measures of quality control (see Materials and Methods). The median *d_N_*/*d_S _*for these 29 genes (*d_N_*/*d_S _*= 0.27, Q1-Q3 = 0.16-0.49) was significantly higher than the median estimated *d_N_*/*d_S _*for the 429 non-overlapping genes (N = 303 of 429 non-overlapping genes with quality one-to-one orthologs, median *d_N_*/*d_S _*= 0.06, Q1-Q3 = 0.02-0.14) (Wilcoxon Rank Sum Test [WRST] W = 7,336, P < 10^-8^) (Figure 2). In addition to these sequence-based metrics, the 54 overlapping genes had fewer one-to-one orthologs between mouse and rat compared to the non-overlapping genes (29/54 vs. 303/429, respectively[http://www.ensembl.org, version 48], Fisher's Exact Test P < 0.02). This result suggests these genes are evolving so rapidly that orthology is difficult to detect, that they undergo more gene conversion which obscures orthology, and/or that they experience higher rates of gene birth and death.

These patterns of rapid evolution derived from mouse-rat comparisons were robust to the precise set of non-overlapping genes investigated. All patterns remained statistically significant even if we compared the 54 overlapping genes to the 88 (of 429) non-overlapping genes that i) have a one-to-one ortholog found in human ejaculates [83], and ii) have a one-to-one ortholog in rat. These additional comparisons represented an attempt to control for possible protein contamination, and to focus on those proteins that show the most evidence of being ejaculated [following, 49].

Unfortunately, we cannot perform deeper evolutionary analyses for most of these genes because orthology across the five mammalian genomes analyzed here (mouse, rat, dog, human, cow) is lacking. It is possible that rapid evolution has obscured orthology assignment. Similar patterns have been observed in insects [92]. Of the 54 overlapping proteins, only 15 have orthologs across the five species, which is a significantly smaller proportion than the 216 (of 429) non-overlapping proteins that have orthologs across the five species (FET, P = 0.001). Of the 15 overlapping proteins with orthologs, two showed statistically significant evidence of adaptive evolution according to the five criteria above (*tissue inhibitor of metalloproteinase 1 *and *plasminogen activator urokinase*), which was not significantly different than the 17 adaptively evolving genes identified from the 216 non-overlapping proteins with orthologs (FET, P = 0.36). Attempts to gain power by analyzing more closely related genomes of rabbit, guinea pig, kangaroo rat, and squirrel (http://www.ensembl.org) were inconclusive due to the low coverages of these additional genomes (data not presented).

Of the 69 ejaculated proteins detected in the present study, 15 were not observed in our previous analysis of the male reproductive tract (Figure 3). These proteins may derive from regions of the male reproductive not sampled in our previous study, for example the ampullary gland, a small swelling in the vas deferens. It is also possible some of these 15 proteins were more easily detected after ejaculation into the female reproductive tract. These 15 proteins evolved at a rate similar to the 54 overlapping proteins (Figure 3).

### Rapid evolution of female-derived endopeptidases, male-derived endopeptidase inhibitors, and copulatory plug genes

Female-derived endopeptidases and male-derived endopeptidase inhibitors evolve relatively rapidly, although our study is underpowered given the low number of genes in both categories. In pairwise mouse-rat estimates, the female-derived endopeptidases *lactotransferrin *and *kallikrein related peptidase 14 *showed a *d_N_*/*d_S _*of 0.78 and 0.32, respectively, values that are substantially higher than the genome of median 0.13. Furthermore, *lactotransferrin *showed statistically significant evidence of recurrent positive selection across a phylogeny of five mammalian species (according to five criteria discussed previously, 1: the data fit the M8 model significantly better than M7 [2ΔL = 29.8, P < 10^-6^], 2: the data fit the M8 model significantly better than M8a [2ΔL = 22.9, P < 10^-5^], 3: the additional class of *d*_N_/*d*_S _estimated by M8 = 3.8, 4: an estimated 4.9% of codons belonged to this additional class, and 5: FEL analyses estimated that 2% of codons experienced *d*_N_/*d*_S_>1.1 at P < 0.10). Only three male-derived endopeptidase inhibitors - *cystatin C, spink5*, and *timp1 *- had high quality orthologs between mouse and rat, but all three showed high *d_N_*/*d_S _*of 0.41, 0.49, and 0.52, respectively. *Timp1 *showed statistically significant evidence of recurrent adaptive evolution across the five mammalian species (1: 2ΔL = 9.81, P < 0.01, 2: 2ΔL = 4.82, P < 0.03, 3: additional class of *d*_N_/*d*_S _= 2.9, 4: estimated 4.9% of codons belonged to this class, and 5: FEL estimated 1.4% of codons with *d*_N_/*d*_S_>1.1), *spink5 *did not, and *cystatin C *could not be analyzed due to a lack of orthology. Rapid evolution of female-derived endopeptidases and male-derived endopeptidase inhibitors is consistent with a model of sexual conflict between these two molecular classes [93,94], though additional functional experiments are required to evaluate this hypothesis further.

Proteins involved in the formation of the copulatory plug showed especially rapid evolution. Four genes known to form a large proportion of the copulatory plug - *SVS1, SVS2, SVS5*, and *Tgm4 *(the other *SVS *genes drop out of pairwise mouse-rat comparisons due to either lack of orthology or failed quality control) - have *d_N_*/*d_S _*estimates of 0.36, 0.40, 0.67, and 0.33, respectively, which are approximately three or more times the genome median (0.13).

## Discussion

A major finding over the past ~15 years is that male reproductive proteins diverge rapidly in sequence [reviewed by 95], gene birth/death processes [96-99], expression [100-103], and protein size or composition [104-107]. Adaptive evolution of copulatory plug proteins is especially strong in species with relatively high levels of polyandry [106,108-110]. In primates, copulatory plug proteins also show signs of rapid evolution [111,112], and the solidification intensity of the plug is positively correlated with the level of sperm competition [39]. In *Drosophila*, both male- and female-derived proteases have undergone rampant duplication, gene conversion, and/or adaptive evolution [93,113-115]. There are several hypotheses to account for this elevated rate of divergence, including adaptive evolution related to natural selection and/or intra- or inter-sexual selection. Disentangling these alternative hypotheses requires a better understanding of the function of ejaculated proteins. Here we used isotopic labeling to separate female- from male-derived proteins taken from the female reproductive tract, identifying 69 proteins that are transferred during mating.

Two functions - *antioxidant activity *and *endopeptidase inhibitor activity *- were significantly enriched among the 69 identified proteins. Sperm are particularly susceptible to oxidative stress as a result of their high metabolic rate, their high level of polyunsaturated fatty acids in their membranes, and their lack of most cytoplasmic components of the antioxidant system. Oxidative stress can damage the paternal genome, leading to aberrant embryonic development [116]. Male hamsters that had their accessory glands surgically removed ejaculated sperm with elevated DNA damage compared to sham-operated controls [117]. In humans, sub-fertile men had a higher level of reactive oxygen species and lower antioxidant ability in their seminal fluid, compared to normally fertile men [118]. In some birds, more colorful males harbor sperm that are more resistant to oxidative stress, raising the possibility that males advertise their ability to protect sperm [119].

Male seminal fluid was also significantly enriched for proteins with *endopeptidase inhibitor *activity. Such proteins are involved in a diversity of physiological functions including modulation of immune response and sperm capacitation. Dean et al. [49] hypothesized that endopeptidase inhibitors may protect the copulatory plug from degradation.

On the female side of the equation, two of the six identified female-derived genes, *lactotransferrin *and *kallikrein related-peptidase 14*, included domains indicative of endopeptidases. One possible function for female-derived endopeptidases is the degradation of the copulatory plug [49]. While there is some reference in the literature to the plug "falling out" or being easily dislodged by females or other males [56], in our extensive experience with wild-derived mice (like those of the present study), the plug is strongly attached to the tissues of the vagina and cervix, rarely visible externally, and requires considerable effort to dissect. Female-derived endopeptidases might degrade the plug and/or detach the plug from its close association to female tissue as an initial step in dislodgement.

Female-derived endopeptidases might be targeted by male-derived endopeptidase inhibitors. Of the six male-derived endopeptidase inhibitors identified above, three were characterized as I4 subfamily members and two as I1 subfamily members [the sixth is not characterized, merops.sanger.ac.uk 120]. Members of subfamily I1 are known to inhibit endopeptidases of the S1 family [121], like the female-derived *kallikrein related peptidase 14 *that we identified here. The other female-derived endopeptidase that we identified, *lactotransferrin*, is part of the S60 family of endopeptidases, which is not known to be inhibited by any of the male-derived endopeptidase inhibitors identified here [120]. More direct experiments are needed to test whether female-derived endopeptidases and male-derived endopeptidase inhibitors interact directly.

Curiously, an additional 429 proteins previously identified in the male reproductive tract by Dean et al. [49] were not observed here. We consider three hypotheses to explain why we did not identify these 429 non-overlapping proteins in this study. One hypothesis is these 429 non-overlapping proteins were not ejaculated. Our earlier work was based on tissue dissection and may therefore have included some contamination by non-ejaculated proteins. This hypothesis seems unlikely to be the main explanation because 327 of the 429 non-overlapping proteins had a one-to-one ortholog in humans, and of those, 114 were detected in human ejaculates [83]. We note that the general findings in either study were not altered if we confined analyses to those genes that had a one-to-one ortholog to a human-ejaculated gene.

A second hypothesis is that even though female proteins were labeled with heavy nitrogen, their presence still reduced the signal-to-noise ratio at various stages throughout the mass spectrometry pipeline employed here. This hypothesis also seems unlikely because technical replication (Figsures 1,2) as well as three independent targeted searches (see *Evaluating Detection Sensitivity *in Results) all suggested we have identified most detectable proteins. Because we used the same mass spectrometry techniques in both studies and the same mouse genotype, the 429 non-overlapping proteins should have been detected if present, unless they were post-translationally modified in ways that make them undetectable only after ejaculation. Other technical artifacts associated with mass spectrometry, such as random loss of signal due to precise composition of co-eluting molecular species, predict a random subset of genes would be identified in our heavy isotope framework, which was not observed here.

A third hypothesis is that many of the 429 non-overlapping proteins were degraded in the female reproductive tract after ejaculation but prior to our sampling of female reproductive tracts. Wild-derived mice demonstrate complicated mating behaviors, so sampling female reproductive tracts immediately after ejaculation is difficult. Thus, for these initial experiments, female reproductive tracts were sampled 6-14 hours after copulation. During this interval, changes in the number and relative abundance of male proteins may have occurred. Consistent with this hypothesis, females produced endopeptidases in response to mating, which may actively degrade ejaculated proteins. Under this scenario, male proteins might be under selection to evolve rapidly, thus evading female degradation machinery. The 69 ejaculated proteins indeed evolved significantly more rapidly than other male reproductive proteins.

## Conclusion

We applied isotopic labeling to directly identify 69 proteins transferred from males to females during mating. The techniques applied here make it possible to study the fate of ejaculated proteins over time. Future experiments can use targeted proteomic methods to follow *in vivo *the localization and degradation of specific male proteins in the female reproductive tract, to more fully appreciate their roles in reproduction and evolutionary fitness.

## Authors' contributions

MDD conceived of the study, designed and performed the crossing experiments, analyzed all data, and wrote the manuscript. GDF helped conceive the study, designed and performed protein isolation and mass spectrometry experiments, and helped write the manuscript. MRH modified algorithms to evaluate peptide labeling and perform directed peptide sampling, and helped perform targeted mass spectrometry experiments. CCW provided heavy nitrogen chow and helped design experiments. MJM helped design experiments and oversaw all mass spectrometry experiments. WJS contributed to experimental design and data interpretation. MWN designed experiments, interpreted data, and contributed to the overall study. All authors have read and approved the final manuscript.

## Supplementary Material

Additional file 1 **Male-derived genes detected in the female reproductive tract**. The 69 genes that code proteins transferred from males to females. Click here for file

Additional file 2 **Ambiguous male-derived genes**. 30 genes that were only identified with ambiguously mapping spectra from the female reproductive tract. Click here for file

Additional file 3 **Genes detected from the male reproductive tract**. 483 genes identified from dissected regions of the male reproductive tract [a re-analysis of 49]. Click here for file
